# Combined anteroposterior approaches in lateral position treatment of lumbosacral tuberculous in single-stage

**DOI:** 10.1186/s12893-022-01612-0

**Published:** 2022-04-29

**Authors:** Jianqiang Bai, Qun Xia, Jun Miao

**Affiliations:** 1 Department of Spine Surgery, Tianjin Hospital, 406 Jiefang Nan Road, Hexi District, Tianjin, 300211 China; 2 Department of Orthopedics, No. 1 Medical Center of Tianjin Hospital, Tianjin, 300211 China

**Keywords:** Lumbosacral tuberculosis, Single-stage, Lateral position anteriorposterior approaches

## Abstract

**Background:**

The local anatomy of the lumbosacral region of spine is complicated, with special biomechanical characteristics. For surgical management of tuberculous spondylitis reported in the literature, the methods would be two-stage anterior and posterior approaches or one-stage anterior and posterior approach with patient’s intraoperative position being changed. These types of surgery approaches would result in long operative duration and more intraoperative blood loss, and most important there is no coordination between anterior and posterior procedures.

**Methods:**

The purpose of this study was to introduce a new procedure called in the lateral position single -stage combined anteriorposterior approaches for treatment of lumbosacral tuberculous spondylitis and to evaluate its preliminary surgical outcomes. Fifteen patients with lumbosacral tuberculous spondylitis who underwent single-stage anterior and posterior radical focal debridement and reconstruction in lateral position in our hospital from April 2005 to June 2012 were included in the study. There were 6 males and 9 females with the average age of 46.8 years. The tuberculous lesions involved the following regions: L3-4 in 5cases, L4-5 in 5 cases, L5-S1 in 2 cases, L4 in one case, and L5 in 2 cases. The assessment of surgical outcomes was conducted with clinical symptoms and radiological findings,including operative time, blood loss. deformity angle, Frankel grade and Kirkaldy-Willis evaluation.

**Results:**

Operation posture: The right lateral position was used for 11 patients and the left lateral position was used for the remaining 4 patients. The average duration of operation for the 15 patients was 270 min. The average intraoperative blood loss was 1720 ml. The mean follow-up period was 4.2 years. There was no recurrence. The postoperative radiological findings showed that the interbody bone grafts were fixed without any dislodgment. There was significant difference between preoperative and postoperative lumbosacral lordotic angles. Kirkaldy-Willis classification rating for the 13 cases with satisfactory results.

**Conclusion:**

Single-stage combined anterior and posterior surgical management of lumbosacral tuberculous spondylitis with patient in lateral position can achieve radical focal debridement, anterior and posterior procedure coordination and spinal reconstruction.

## Background

Recent years the incidence of tuberculosis is rising. Tuberculous spondylitis is the most common form of osteoarticular tuberculosis [1]. In The conservative treatment of antituberculous chemotherapy and external bracing remain the first choice of treatment for tuberculous spondylitis [2, 3]. Surgery is recommended in the presence of significant spinal deformity and spinal cord compression symptoms due to severe vertebral destruction. The lumbosacral portion is a transitional region which connects the spine to the pelvis. The local anatomy of this portion of spine is complicated, its biomechanical characteristics are special. Tuberculous spondylitis of lumbosacral region only accounts for 2% to 3% of spinal tuberculosis [4]. Its surgical treatment has rarely been documented in the literature [5] and no standard surgical approach has been established, with the options including anterior, posterior, and combined anterior and posterior approaches [6]. Anterior or posterior approach alone will not provide good exposure of the involved vertebrae. Therefore, the resection of tuberculous lesion and fusion with bone graft can’t be achieved satisfactorily [7]. The traditional combined anterior and posterior approach either requires two-stage operation or patient’s position needs to be changed and skin needs to be asepticized again during surgery. A major disadvantage for both methods is that the anterior and posterior procedure are performed separately, unable to cooperate with each other. For example, if the posterior fixation has been finished, then the anterior interbody bone graft cannot be expanded and clasped in an optimal way. And if the anterior strut bone grafting is performed first, then there is a chance that the bone graft will be moved when the patient’s position is being changed, which would result in spinal cord compression [8].

The purpose of this retrospective study was to introduce a new procedure called in the lateral position single -stage combined anteriorposterior approaches for treatment of lumbosacral tuberculous spondylitis. We evaluated the clinical outcomes of this procedure for treatment of 15 patients with lumbosacral tuberculous spondylitis. The following are the details of this study.

## Methods

### Patient population

All fifteen patients (6 males and 9 females) with lumbosacral tuberculous spondylitis who were hospitalized and treated in our department from April 2005 to June 2012 were included. The average age was 46.8 years (range, 24–65 years). All the 15 patients experienced low back pain of variable severity which would be aggravated by motion, and all of them had the typical symptoms of tuberculosis infection such as low-grade fever, night sweating, fatigue, etc. Tuberculous lesion involved the following regions: L3-4 in 5 cases, L4-5 in 5 cases, L5-S1 in 2 cases, L4 in one case, and L5 in 2 cases. The time interval between the onset of symptoms and surgery was 7 months on average (range, 5–12 months). The radiographs mainly demonstrated severe vertebral destruction, bone sequestration and intervertebra space narrowing. Of these 15 patients, 13 had lesions involving spinal canal, while only 7 of them had the symptoms of cauda equina compression. According to the Frankel classification, the neurological function was grade C in 2 cases, grade D in 8 cases and grade E in 5 cases[9].

### Preoperative preparation

Preoperative erythrocyte sedimentation rate (ESR) and C-reactive protein (CRP) level were increased in all the 15 cases, and the average ESR was 65 mm/h (range, 40-106 mm/h). Moderate anemia and low protein level occurred in 2 cases. Chest X-ray examination was performed for all the patients preoperatively in order to exclude acute miliary pulmonary tuberculosis. The standard four-drug anti-TB regimen with including isoniazid (5 mg/kg), rifampicin (10 mg/kg), ethambutol (15 mg/kg), and pyrazinamide (25 mg/kg) was administered for over 3 weeks. The surgery was performed when ESR < 40 mm/h or significantly decreased, and hemoglobin (Hb) > 10 g/L. Intraoperative lesion biopsy was performed for pathological evaluation to further confirm the diagnosis.

### Surgical technique

All the 15 patients underwent surgery under general anesthesia.

#### Position

**Fig. 1:**
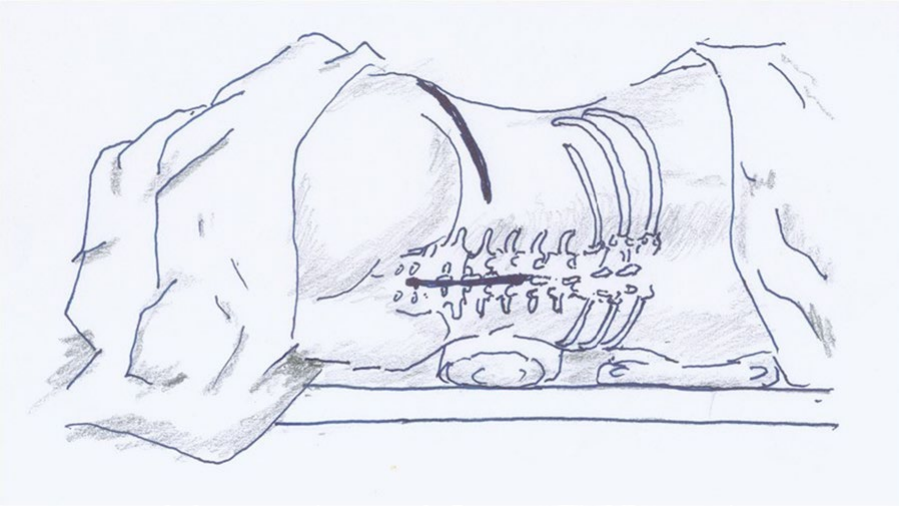
Schematic diagram of surgical position and incision

Lateral position was adopted and the regions of psoas abscesses were considered as a determinant in choosing the left or right lateral position. The right lateral position was used for 11 cases including 8 cases of left-side psoas abscess and 3 cases of bilateral psoas abscess with the left one being more severe. The left lateral position was used for the remaining 4 cases with right-side psoas abscess. Pads are required under the waist. It is necessary to keep the hip and knee flexed (Fig. 1).

#### Posterior approach

The posterior approach procedure was performed first. The operating table was tilted down laterally to the patient’s back side at a angle of 30 to facilitate surgeon posterior operation. A midline straight skin incision and blunt dissection were performed to expose the laminae and the facet joints on both sides. Pedicle screws were then inserted into the vertebral bodies above and below the affected one. (Pedicle screw fixation was performed on the bilateral ilia for patients whose L5-S1 intervertebral disc was severely damaged and the lesion involved S1 vertebral body.) A connecting rod was bent in accordance with the natural physiological curvature of lumbosacral portion and fixed temporarily. Kyphotic deformity was corrected by moderately expanding the pedicle fixation system No decompressive laminectomy was performed and the normal vertebral lamina remained intact. When all these procedures were finished, gauze packing was used for hemostasia and the posterior approach incision was closed temporarily to keep the operative field relatively sterile.

#### Anterior approach

Then the anterior approach procedure was performed. The operating table was adjusted back to be in the horizontal position. An abdominal oblique incision was centered on the involved vertebra. The anterior approach was made through external oblique muscle, internal oblique muscle, and transversus abdominis muscle into extraperitoneal space. The peritoneum and the contents in the abdominal cavity were slightly pushed to the front with blunt tools. The greater psoas was longitudinally divided. Then the psoas abscess was drained and the caseous matter was removed. The side aspect of infected vertebra was exposed and the blood vessels which supply the region were cut off after ligation. The infected vertebra was completely exposed and the involved intervertebral disc was resected, leaving the normal bony endplates intact. Using osteotome and hand bone curette radical resection of infected vertebra was performed until healthy or sub-healthy bone was exposed. For cases with cauda equina compression, the posterior longitudinal ligament was cut to facilitate the removal of abscess and necrotic matter from inflammatory reaction in the spinal canal for complete decompression. After radical resection of tuberculous lesion, the resultant gap was measured and a piece of titanium mesh of appropriate length was prepared. The titanium mesh was filled with cancellous bone allograft and then inserted into the gap with the bone graft supported by the titanium mesh, or an appropriate autogenous iliac bone was inserted into the gap. The titanium mesh was trimed to form a bevel to fit the interface between the mesh and the upper endplate of sacrum in order to accommodate the lumbosacral angle.

#### Combined anteriorposterior

**Fig. 2:**
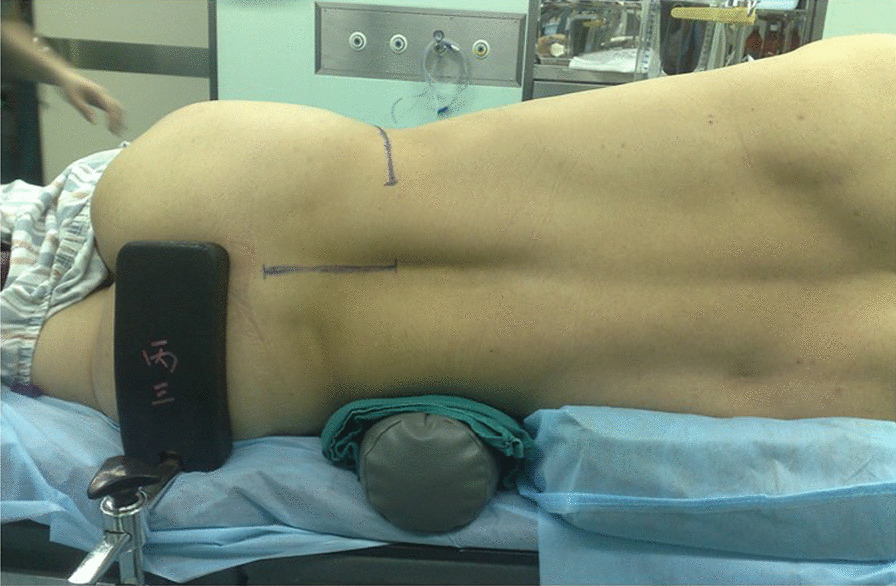
Intraoperative actual surgical position and incision

Then the anterior and posterior procedures were operated coordinately (Fig. 2). The sterile gloves and medical devices were replacement and gone into rear incision once again. Then the implanted pedicle fixation system was longitudinal compressed moderately in order to clasp the anterior strut bone graft to prevent it from loosening or dislodgment. Assistant used hemostat from the front clamp titanium mesh and shake test the stability of held satisfactory.and then the screw caps were tightened and a cross-linkage was placed. Streptomycin (1 g) was used in the infected focus as a routine treatment before the closure of the anterior approach incision. A drainage tube was placed in each surgical wound and an abdominal binder was used after surgery.

### Postoperative care

Ambulation was allowed 2 weeks after surgery with the protection of lumbar orthosis and a lumbar orthosis was worn by the patient for 3 months. Antituberculous chemotherapy was continued for a year after surgery using the four ral antituberculous drugs rimifon (5 mg/kg), rifampicin (10 mg/kg), ethambutol (15 mg/kg), and pyrazinamide (25 mg/kg).

### Postoperative evaluation

Antero-posterior and lateral radiography of lumbar vertebrae, ESR test, and for some cases three-dimensional reconstruction of CT scan data or MRI scan were performed at 3 months, 6 months and final follow up after surgery. The lordotic angle of lumbar vertebrae is the angle formed from the upper end-plate of L1 to the upper end-plate of S1 pre-operatively, post-operatively and at the final follow-up. The measurement of lumbosacral lordotic angle and the assessment of spinal fusion were conducted by two spine surgeons. The neurological function was evaluated with the Frankel neurological classification [9]. The functional outcomes were determined using the Kirkaldy-Willis criteria [10], and the class of excellent or good meant the result was satisfactory. Fusion was assessed by the radiological findings, according to the criteria established by Lee et al. in 1995 [11].

### Statistical analysis

Comparison of the angle of lumbar lordosis after before surgery, immediate period, 6 months and 1 year after after surgery using analysis of variance, p < 0.05 as significant difference. The software SPSS 22 0.0 was adopted for the statistical analysis. Fusion was assessed using the criteria of Lee, Vessa and l.ee" (Table 1). Functional outcome was graded using the neurological classification of Frankel and the functional criteria of Kirkaldy-Willis. A satisfactory outcome included or excellent and good result (Table 1).

**Table 1:** Summary of the patient demographics, operative information, and follow-up period

n	Age/Gender	Involved segment	Frankel Score		Deformity Angle (°)				Bleeding amount (mL)	Operation Time(h)	Following time (Y)	Kirkaldy-Willis
			Preop	Late	Preop	Postop	1.5 Y	Fin				
1	47/W	L4	D	E	7.8	18.1	15.5	15	2700	5.5	4.7	Excellent
2	53/W	L3 L4	E	E	27.5	37.2	35.5	34.5	3000	5.0	4.5	Good
3	65/M	L4 L5	C	E	23.5	34.3	33.2	31.9	1800	4.0	3.5	Good
4	24/M	L4 L5	D	E	20.0	31.5	30.3	29.9	1100	4.3	2.2	Excellent
5	37/W	L3 L4	D	E	23.6	30.3	29.5	28.5	1400	4.4	3.8	Excellent
6	65/W	L3 L4	E	E	21.9	33.7	33.1	31.7	1600	5.2	5.5	Good
7	29/W	L4 L5	E	E	19.0	28.8	26.8	25.3	1400	4.0	5.2	Excellent
8	27/M	L3 L4	D	E	22.3	35.3	33.2	32	1200	4.1	5.5	Excellent
9	51/W	L5 S1	E	E	22.1	31.1	26.3	26	1300	5.7	3.5	Good
10	57/W	L4 L5	C	D	23.7	34.3	32.1	32	1900	4.6	4.5	Fair
11	38/M	L5	E	E	14.3	23.1	22.7	19.5	2500	4.0	2.4	Excellent
12	54/M	L3 L4	E	E	21.6	31.4	29.5	29	1800	4.3	2.6	Excellent
13	38/W	L5	D	D	13.2	24.1	22.2	21.5	1400	3.6	6.7	Fair
14	61/W	L4 L5	E	E	25.0	34.9	32.0	32	1200	4.2	5.3	Excellent
15	56/M	L5 S1	E	E	21.7	30.1	28.1	27.5	1500	4.8	3.2	Good
Ave	46.8				20.5	30.5	28.6	27.7	1720	4.5	4.2	

## Results

### Clinical results

**Fig. 3:**
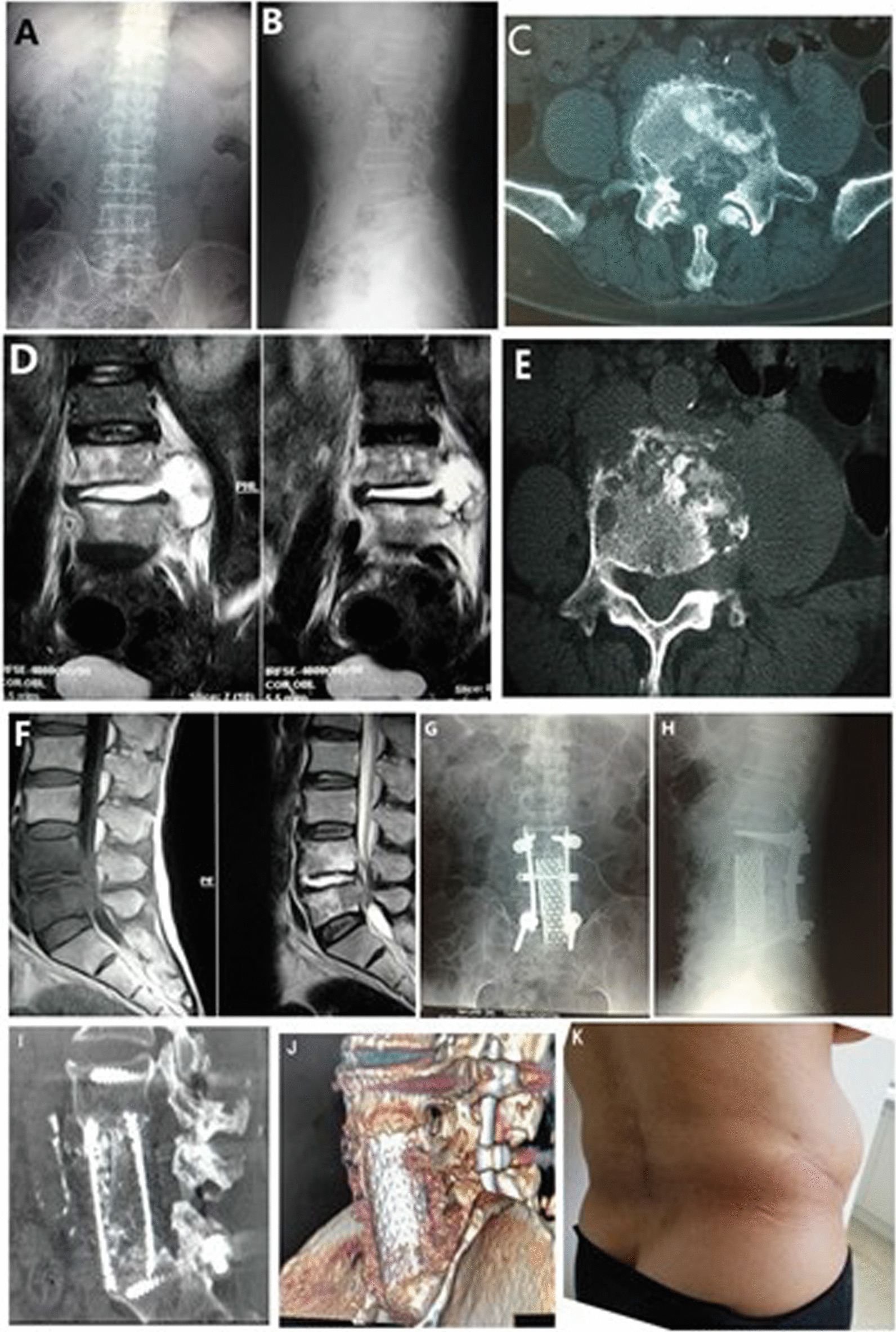
A 65-year-old man L4-5 TB. Preoperative, posteroanterior **A** and lateral **B** plain radiographs. Cross-sectional CT scan **C**, **E** and MRI **D**, **F** views showed bony destruction form the L4–L5 vertebrae, with prevertebral and paravertebral abscessed compressing the neural elements. Postoperative radiographs include anteroposterior **G** and lateral (**H**). The last folloe-up 3D CT showed good fusion (**I**, **J**). Photo of the patient’s incision at the last review (**K**)

The surgical duration for all the 15 patients was 270 min on average (range, 220–380 min). The average intraoperative blood loss was 1720 ml (range, 1100–3000 ml). The average duration of bed rest was 14 days. All the patients achieved primary healing for surgical wounds and no infection or local sinus formation occurred. The mean follow-up period was 4.2 years (range, 2.2–6.7 years). The average ESR decreased to 15 mm/h at 6 weeks after surgery and returned to normal at 6 months after surgery. The significant relief of low back pain and muscle spasm was achieved after surgery in all the 15 patients. Thirteen patients didn’t experience any low back pain during daily activity, while two still complained of discomfort in the lumbosacral region at the final follow up. The neurological function was improved significantly. According to the Frankel classification, 2 cases were of grade D and 13 cases were of grade E. With the Kirkaldy-Willis criteria, the functional outcomes were classified as excellent in 8 patients, good in 5 patients, fair in 2 patients, and there was no poor case (Fig. 3).

### Complications

One patient had mild hemorrhagic shock and recovered after blood transfusion. Two patients experienced transient abdominal distension after surgery. One patient suffered superficial abdominal wound infection and recovered by changing dressings.

### Imaging results

**Fig. 4:**
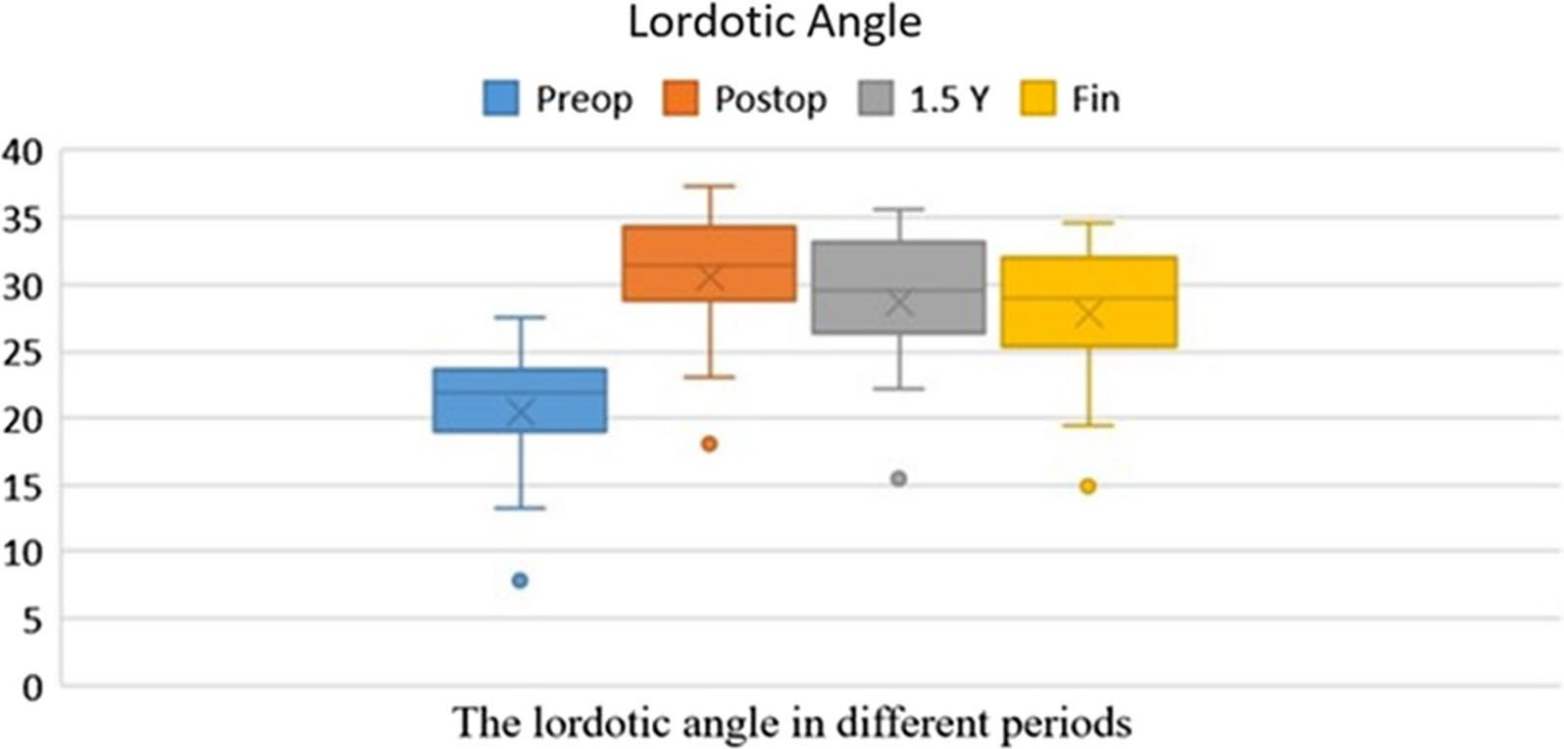
The lordotic angle in different periods. There was a significant difference between preoperative with postoperative, 1.5 years and the final follow up lordotic angles of lumbosacral portion (p < 0.05)

There was no recurrence at the final follow up, and the bone grafts were fixed without any loosening or dislodgment. The preoperative average lordotic angle of lumbosacral portion was 20.5° ± 5.1°, the postoperative one was 30.5° ± 5.2°, 1.5 years was 28.6° ± 5.7°, and the one at the final follow up was 27.7° ± 5.4°. There was a significant difference between preoperative and postoperative lordotic angles of lumbosacral portion (P < 0.05). There was no significant difference between the postoperative lordotic angle,1.5 years, and the final follow up (P = 0.31,0.28). There was a significant difference between the preoperative lordotic angle with 1.5 years and the one at the final follow up (P < 0.05) (Fig. 4). Fusion assessment: The CT scan for all the patients were examined to assess the fusion at the final follow-up. All patients achieved bone fusion. According to the classification of Lee et al.[11], 14 patients achieved complete fusion (Grade A), whereas only 1 patients achieved partial fusion (Grade B).

## Discussion

### The features of lumbosacral tuberculous spondylitis

There have particular anatomical feature in Lumbosacral region. It is located between lumbar vertebrae with larger activities and sacral vertebra which is relatively fixed, playing a role as transition structure. The vessels located in front of the lumbosacral vertebrae include abdominal aorta, inferior vena cava, common iliac arteries, veins and middle sacral artery, vein. The lateral sides of the lumbosacral vertebral segment are obstructed with alae of ilium. Therefore, the local anatomy of this region is complex which leaves limited operation space for surgeons. The tuberculous focus at this region is hard to be operated [5]. The blood supply of this portion is poor than other parts of the spine. When tuberculous infection occurs, the tubercle bacilli loaded in blood stream will be deposited at this region due to the low rate of blood flow [2]. The lumbosacral vertebrae also bear higher shear force and pressure stress and are comparatively mobile [12]. Therefore, the infection of tuberculosis at this site will often be localized and the healing will be delayed.

The spinal canal will be narrowed when vertebral destruction, collapse and the formation of paravertebral abscess occur which need surgical management quickly [13]. Although spinal canal involvement occurred in 93% of the patients in this study, only 13% of the patients demonstrated the symptoms arising from severe cauda equina damage because of the relatively large size of the spinal canal of lumbosacral portion [6]. The destruction of lumbosacral vertebrae is generally demonstrated as a flattening of the normal lordosis and kyphotic deformity is rarely observed due to the natural lordotic structure of the physiological lumbosacral vertebrae [14]. The preoperative lordotic angle for the patients in this study was 20.5° on average, which was consistent with the previously reported ones [2, 6]. The goals of treatment of lumbosacral tuberculous spondylitis are not only to eradicate the infection but also to restore the spinal stability of this portion. Researches indicate that on the basis of strict chemotherapy regimen, only when spinal stability is effectively maintained can tuberculosis be cured completely [15]. Since the advent of internal fixation technique in the mid-1990s, the therapy for spinal tuberculosis has been led into a new period of development. The internal fixation technique provides the immediate reconstruction and maintenance of spinal stability; it facilities spinal fusion with bone grafting; it can even correct kyphotic deformity. Overall, with this technique, the goal of early ambulation can be achieved. Some studies demonstrate that the safety of using the internal fixation system in the treatment of spinal tuberculosis should be attributed to the low adherence of tubercle bacilli to the implant materials [16]. In addition, the biofilms formed by tubercle bacilli around the internal fixation devices are less than those formed by other bacteria, which might also contribute to the safety of using the internal fixation system [17].

### The options of surgical approaches for lumbosacral tuberculous spondylitis

The surgical approaches are mainly anterior, posterior, and combined anterior and posterior approaches. Lee et al. reported 16 cases with the treatment of posterior decompression and pedicle screw fixation for lumbar tuberculous spondylitis [18]. This operative method is suitable for treating early-stage tuberculosis with mild bone destruction or with infection only involving the posterior column. With the posterior approach surgery, direct contact with tuberculous lesion can be avoided, which will reduce the dissemination of infection, and the spinal deformity can be corrected to some extent [19]. While lumbosacral tuberculous spondylitis generally will not involve the spinal posterior column. Single posterior approach surgery will damage the integrality of posterior column, resulting in worsening of instability because the anterior column have already been damaged. If resection of infected vertebra and placement of titanium mesh were performed from posterior approach anyway, the roots of important nerves innervating the lower limbs may be injured. Moreover, the treatment goals of radical resection of tuberculous lesion and removal of psoas abscess can’t be achieved. Therefore, this method has rarely been used in clinic[20]. We had only found one report from Bezer et al. about the surgical method of transpedicular drainage, posterior instrumentation and interbody bone grafting for the treatment of lumbosacral tuberculous spondylitis in 7 patients [5]. However, the anterior focus hadn’t been completely removed. This method is inapplicable for the cases of severe vertebral destruction and cauda equina compression. Anterolateral approach surgery for only removal of focus of infection is a traditional method for operation of tuberculous spondylitis. With this approach, the abscess, caseous matter and sequestrated bone could be removed. The disadvantages are: the exposure of tuberculous lesion is limited; the resection of infected vertebra is incomplete; no internal fixation is used for strut bone graft; the stabilization of lumbosacral region can’t be achieved immediately; bone graft absorption, subsidence or dislodgment are prone to occur; the lumbosacral angle will be reduced [21]. To prevent the occurring of these adverse events, plaster beds are used for the immobilization of patients for several months after surgery, which makes nursing care procedures difficult to perform and also is unbearable for the patients. With the advent of advanced medical techniques, anterolateral approach surgery of lateral fixation systerm has been introduced. It can achieve the goals of radical resection of tuberculous lesion and strut bone grafting. The lateral fixation system can prevent titanium mesh or bone graft from dislodgment and reconstruct the anterior and middle columns of spine. Hodgson and Stock adopted this surgical method for the treatment of spinal tuberculosis and achieved a cure rate of 89.9% [22]. This method was called as “Hong Kong operation” in the medical field. While for the surgical management of lumbosacral tuberculosis, the pedicle screw fixation at the site distal to L5 vertebra will be hard to perform due to the obstruct of ala of ilium. Therefore, the lateral fixation will be very difficult, especially in patients with damaged S1 vertebra. Thus the lateral approach surgery is not an appropriate method for the treatment of lumbosacral tuberculous spondylitis either. Overall, with single anterior approach, it is impossible to perform the internal fixation due to the obstruct of ala of ilium, and with single posterior approach, it is hard to remove the infected material completely and carry out the strut bone grafting because of the special features in anatomy and biomechanics of lumbosacral region.

Combined anterior and posterior approach is suit for surgical treatment of lumbosacral tuberculous spondylitis [23]. With this method, the reconstruction of the three spinal columns can be achieved which was consistent with the biomechanical principle of the spinal column and was beneficial for patients’early and functional rehabilitation.Even recurrence occurs, there is no need to remove the posterior fixation system. Two types of combined anterior and posterior approach have been reported for surgical treatment of lumbosacral tuberculous spondylitis. One is two-stage anterior and posterior surgery [24]. It needs the surgical treatment to be performed with two separate procedures. Therefore, patients have to undergo general anesthesia twice, the duration of hospital stay will be longer, and the treatment cost will be increased. In the report of Chen et al. 5 of 32 patients with lumbosacral tuberculous spondylitis underwent the two-stage anterior and posterior surgery [8]. The other one is single-stage two-position anterior and posterior surgery. The patient’s position needs to be changed during surgery. This method can reduce the intraoperative blood loss, shorten the duration of hospital stay and lower the treatment cost [21, 25]. Posterior fixation had been done in the prone position firstly. However, a major disadvantage for both methods is that the anterior procedure and posterior procedure are performed separately, which results in none coordination between the two procedures. For example, if the posterior fixation has been finished, then the anterior interbody bone graft cannot be expanded and clasped in an optimal way. And if the anterior strut bone grafting is performed first, then there is a chance that the bone graft will be moved when the patient’s position is being changed, which would result in spinal cord compression [26]. The above mentioned disadvantages can be conquered using single-stage anterior and posterior approach surgery with patient in lateral position.

### Single-stage anterior and posterior approach surgery with patient in lateral position for lumbosacral tuberculous spondylitis

All the 15 patients with lumbosacral tuberculous spondylitis in this study underwent single-stage anterior and posterior approach surgery in lateral position. The procedures include radical focal debridement [27], anterior placement of titanium mesh containing bone graft, and posterior stabilization using pedicle fixation system [28]. The clinical outcomes for 13 cases were satisfactory. The characteristics of this method include:

(1) Coordination between anterior and posterior procedures can be achieved: At first, the posterior pedicle fixation system is expanded. When the anterior decompression and strut bone grafting have been performed, the posterior pedicle fixation system is then contracted to clasp the anterior titanium mesh or bone graft to prevent loosening or dislodgement. (2) The duration of surgery is short and the incisions are small. The radical resection of tuberculous lesion and reconstruction of lumbosacral region are both performed with the patient in lateral position. There is no need to change the patient’s position, therefore, no need to asepticize the skin for the second time. The surgical duration will be shortened. Lateral pedicle screw fixation of the distal L5 and sacral 1 vertebral bodies will be difficult to perform due to obstruction of the ilium wing. The anterior approach needn`t for lateral fixation and only need exposure of infected vertebra which the range of exposure is smaller than the conventional anterior incision. (3) The posterior pedicle fixation system is only needed to fix the upper and lower adjacent vertebrae of the infected one. This will retain the adjacent vertebrae mobile. The posterior pedicle fixation together with the anterior titanium mesh or strut bone graft accomplish the reconstruction of the three columns of spine. The procedures are consistent with the biomechanical principle of spine, and early ambulation will be achieved for functional exercises. (4) The expansion of pedicle fixation system through posterior approach will significantly increase the lordotic angle of lumbar vertebrae, which will help restore the normal physiological curvature of lumbar vertebrae. But lumbosacral tuberculous spondylitis will cause soft tissue adhesion and formation of scar tissue, therefore, the posterior pedicle fixation system should only be expanded moderately. (5) After anterior radical debridement of infected material and strut bone grafting are finished, and the sterile gloves and medical devices are replaced, the posterior clasping of bone graft with forces will be performed. The posterior internal fixation region should be kept relatively sterile in order to reduce infection rate.

This simultaneous anterior and posterior approach surgery for lumbosacral tuberculous spondylitis has integrated the advantages of anterior approach, posterior approach, and two-stage anterior and posterior approach surgeries and make up for their shortcomings.

This retrospective observational study has some limitations. First, the main drawback of our study is a small sample size. Second, a long-term follow-up is lacking in this study. Third, this study is a single-center study. Therefore, future research should consider a larger number of patients, a longer follow-up period and Multi-center collaborative research. A prospective experiment can be designed,with the combined anterior and posterior approach in the traditional dual body position as the control group and the current surgical design as the experimental group. Finally, the existing surgical plan can be improved from the original posterior approach to the percutaneous approach or intramuscular approach (Wiltse approach).

## Conclusions

Single-stage anterior and posterior approach with patient in lateral position is a safe and effective method and is useful in the surgical treatment of lumbosacral tuberculous spondylitis. With it, the radical debridement of tuberculous lesion for complete decompression, the reconstruction of the three spinal columns, and the coordination between anterior and posterior procedures can be achieved. The duration of surgery is relatively short, and there is less complications after surgery. Early ambulation can be achieved for functional exercises. There was no recurrence at the final follow up in this study.

## Data Availability

The datasets and materials generated or analyzed during the current study are available from the corresponding author on reasonable request.
